# Supplementary material for: Motivation and preference in isolation: a test of their different influences on responses to self-isolation during the COVID-19 outbreak
Source: R Soc Open Sci. 2020 May 13;7(5):200458. doi: 10.1098/rsos.200458 (PMC7277280; doi:10.1098/rsos.200458)
Supplement: SOM 1 and SOM 2 [file rsos200458supp1.docx]

*Supplementary Table*

Research Plan Set Out in Stage 1 Submission

| **Question** | **Hypothesis** | **Sampling plan (e.g. power analysis)** | **Analysis Plan** | **Interpretation given different outcomes** | **Support by data?** |
| --- | --- | --- | --- | --- | --- |
| Does preference for solitude protect against higher ill-being when in isolation? | 1:1  Preference for solitude would yield a negative correlation with ill-being 1 and 2 weeks later when participants report about time spent alone in the past week. | We aim to be powered (95%, alpha level .05) to detect an effect size as small as f^2^ = .022 for additional variance explained by the main effects of preference and motivation and interaction of these two variables, above and beyond covariates (baseline ill-being, gender, age, levels of in-person social interactions). This conservative effect size f^2^ = .022 represents approximately 2% of variance explained by the main effects and interaction together, after removing the variance explained by covariates.    However, if we assume up to 50% data loss due to attrition across the two time-points with *n* = 400, we can expect to have 95% power to detect an effect size as small as f^2^ = .043. | In two hierarchical linear regression analyses, we will regress later time ill-being on covariates at Step 1, Time 1 preference for solitude at Step 2, and Time 1 identified and external motivation at Step 3.    Step 2 tests Hypothesis 1:1 and 1:2.    Step 3 tests Hypothesis 2:1, 2:2 and 2:3. | If the regression yields negative correlation between preference for solitude and Time 2 and/or 3 ill-being, with *partial r* larger than -.15, we will claim support for Hypothesis 1:1. If the correlation was positive, with *partial r* larger than .15, we will claim partial or full support for 1:2. | No |
| Does preference for solitude reflect symptoms of psychological vulnerability that will manifest during isolation? | 1:2  Preference for solitude might be positively correlated with ill-being in follow-ups. |  |  |  | No |
| Does identified motivation for solitude protect against higher ill-being when in isolation? | 2:1  Identified motivation for solitude will negatively correlate with residual change in ill-being 1 and 2 weeks later, above and beyond Time 1 preference for solitude. |  |  | If the regression yields positive correlation between identified motivation for solitude and Time 2 and/or 3 ill-being, with *partial r* larger than .15, we will claim partial or full support for Hypothesis 2:1. | No |
| Does external motivation for solitude intensify ill-being consequences of isolation? | 2:2  External motivation for solitude will positively correlate with residual change in ill-being 1 and 2 weeks later, above and beyond Time 1 preference for solitude. |  |  | If the regression yields negative correlation between external motivation for solitude and Time 2 and/or 3 ill-being, with *partial r* larger than -.15, we will claim partial or full support for Hypothesis 2:1. | No |
| Does identified motivation determine when preference for solitude protects against the harmful psychological consequences of isolation? | 3:1  If preference for solitude is negatively correlated with ill-being, this will only be the case when identified motivation for solitude is high (estimated at 1 standard deviation (*SD*) above mean) relative to when identified motivation for solitude is low (-1 *SD*). |  | In two hierarchical linear regression analyses, we will regress Time 2 and Time 3 ill-being, in turn, on covariates at Step 1, Time 1 preference for solitude and Time 1 identified motivation at Step 2, and the interaction term of preference for solitude and identified motivation for solitude at Step 3. We will perform simple slope analyses to determine the correlation between preference for solitude and ill-being at +1 SD and -1 SD levels of identified motivation. | If simple slope analysis yields *larger negative* correlation between preference for solitude and ill-being at +1 SD level of identified motivation for solitude, relative to the *negative* preference-illbeing correlation at -1 SD level of identified motivation for solitude, yielding at least .10 difference between the two standardized coefficients, we will claim support for Hypothesis 3:1 | No |
| Does identified motivation attenuate the positive link between preference for solitude and ill-being? | 3:2  If preference for solitude is positively correlated with ill-being, this will only be the case when identified motivation for solitude is low (-1 *SD*) relative to when identified motivation for solitude is high (+1 *SD*). |  |  | If simple slope analysis yields *larger positive* correlation between preference for solitude and ill-being at -1 SD level of identified motivation for solitude, relative to the *positive* preference-ill being correlation at +1 SD level of identified motivation for solitude, yielding at least .10 difference between the two standardized coefficients, we will claim support for Hypothesis 3:2 | No |
| Does the presence of identified motivation distinguish between when preference is adaptive (leading to lower ill-being) or maladaptive (leading to higher ill-being)? | 3:3  Preference for solitude will yield a positive correlation with ill-being for those at -1 *SD* level of identified motivation for solitude. On the other hand, preference for solitude will yield negative correlation with ill-being for those at +1 *SD* level of identified motivation for solitude. |  |  | If simple slope analysis yields *negative* correlation between preference for solitude and ill-being at -1 SD level of identified motivation for solitude, and instead yield *positive* preference-illbeing correlation at +1 SD level of identified motivation for solitude, yielding at least .10 difference between the two standardized coefficients, we will claim support for Hypothesis 3:3 | No |
| Does low external motivation determine when preference for solitude protects against the psychological consequences of isolation? | 4:1  If preference for solitude yields a negative correlation with ill-being, this will only be the case when external motivation for solitude is low (-1 *SD*) relative to when external motivation for solitude is high (+1 *SD*). |  | In two hierarchical linear regression analyses, we will regress later time ill-being on covariates at Step 1, Time 1 preference for solitude and Time 1 external motivation at Step 2, and the interaction term of preference for solitude and external motivation for solitude at Step 3. We will perform simple slope analyses to determine the correlation between preference for solitude and ill-being at +1 SD and -1 SD levels of external motivation. | If simple slope analysis yields *larger negative* correlation between preference for solitude and ill-being at -1 SD level of external motivation for solitude, relative to the *negative* preference-illbeing correlation at +1 SD level of external motivation for solitude, yielding at least .10 difference between the two standardized coefficients, we will claim support for Hypothesis 4:1 | No |
| Does low external motivation attenuate the positive link between preference for solitude and ill-being? | 4:2  If preference for solitude correlates positively with ill-being, this will only be the case when external motivation for solitude is high (+1 *SD*) relative to when external motivation for solitude is low (-1 *SD*). |  |  | If simple slope analysis yields *larger positive* correlation between preference for solitude and ill-being at +1 SD level of external motivation for solitude, relative to the *positive* preference-illbeing correlation at -1 SD level of external motivation for solitude, yielding at least .10 difference between the two standardized coefficients, we will claim support for Hypothesis 4:2 | No |
| Does low external motivation distinguish between adaptive and maladaptive preference for solitude? | 4:3  Preference for solitude will correlate positively with ill-being for those at +1 *SD* level of external motivation for solitude. On the other hand, preference for solitude will yield negative correlation with ill-being for those at -1 *SD* level of external motivation for solitude. |  |  | If simple slope analysis yields *negative* correlation between preference for solitude and ill-being at +1 SD level of external motivation for solitude, and instead yield *positive* preference-illbeing correlation at -1 SD level of external motivation for solitude, yielding at least .10 difference between the two standardized coefficients, we will claim support for Hypothesis 4:3 | No |
