# Supplementary material for: Motivation and preference in isolation: a test of their different influences on responses to self-isolation during the COVID-19 outbreak
Source: R Soc Open Sci. 2020 May 13;7(5):200458. doi: 10.1098/rsos.200458 (PMC7277280; doi:10.1098/rsos.200458)
Supplement: Appendix 1 and Appendix 2 [file rsos200458supp2.docx]

APPENDIX A

|  |  | Total^a^ | | | | | USA (*n* = 366) | | | | | | | UK (*n* = 454) | | | | | | |  |  |  |  |  |  |  |  |
| --- | --- | --- | --- | --- | --- | --- | --- | --- | --- | --- | --- | --- | --- | --- | --- | --- | --- | --- | --- | --- | --- | --- | --- | --- | --- | --- | --- | --- |
|  |  | T1 | T2 | | T3 | | T1 | | | T2 | | T3 | | T1 | | | T2 | | T3 | |  |  |  |  |  |  |  |  |
| **Gender** | |  |  | |  | |  | | |  | |  | |  | | |  | |  | |  |  |  |  |  |  |  |  |
|  | Male | 363 |  | |  | | 181 | | |  | |  | | 182 | | |  | |  | |  |  |  |  |  |  |  |  |
|  | Female | 456 |  | |  | | 184 | | |  | |  | | 270 | | |  | |  | |  |  |  |  |  |  |  |  |
|  | Other gender | 2 |  | |  | |  | | |  | |  | | 2 | | |  | |  | |  |  |  |  |  |  |  |  |
|  | No response | 2 |  | |  | | 1 | | |  | |  | |  | | |  | |  | |  |  |  |  |  |  |  |  |
|  |  |  |  | |  | |  | | |  | |  | |  | | |  | |  | |  |  |  |  |  |  |  |  |
| **Employment status** | |  |  | |  | |  | | |  | |  | |  | | |  | |  | |  |  |  |  |  |  |  |  |
|  | Full-time | 323 |  | |  | | 140 | | |  | |  | | 183 | | |  | |  | |  |  |  |  |  |  |  |  |
|  | Housewife | 2 |  | |  | |  | | |  | |  | | 2 | | |  | |  | |  |  |  |  |  |  |  |  |
|  | Part-time | 99 |  | |  | | 36 | | |  | |  | | 62 | | |  | |  | |  |  |  |  |  |  |  |  |
|  | Retired | 178 |  | |  | | 82 | | |  | |  | | 96 | | |  | |  | |  |  |  |  |  |  |  |  |
|  | Self-employed | 120 |  | |  | | 61 | | |  | |  | | 59 | | |  | |  | |  |  |  |  |  |  |  |  |
|  | Students | 7 |  | |  | | 4 | | |  | |  | | 3 | | |  | |  | |  |  |  |  |  |  |  |  |
|  | Unemployed | 64 |  | |  | | 29 | | |  | |  | | 35 | | |  | |  | |  |  |  |  |  |  |  |  |
|  | Other | 30 |  | |  | | 14 | | |  | |  | | 15 | | |  | |  | |  |  |  |  |  |  |  |  |
|  | Missing | 1 |  | |  | |  | | |  | |  | |  | | |  | |  | |  |  |  |  |  |  |  |  |
|  |  |  |  | |  | |  | | |  | |  | |  | | |  | |  | |  |  |  |  |  |  |  |  |
| **Marital status** | |  |  | |  | |  | | |  | |  | |  | | |  | |  | |  |  |  |  |  |  |  |  |
|  | Divorced | 173 |  | |  | | 95 | | |  | |  | | 78 | | |  | |  | |  |  |  |  |  |  |  |  |
|  | Divorced/Separated/Widow | 28 |  | |  | | 15 | | |  | |  | | 13 | | |  | |  | |  |  |  |  |  |  |  |  |
|  | Living apart but steady | 39 |  | |  | | 10 | | |  | |  | | 28 | | |  | |  | |  |  |  |  |  |  |  |  |
|  | Living together as married | 23 |  | |  | | 11 | | |  | |  | | 12 | | |  | |  | |  |  |  |  |  |  |  |  |
|  | Married | 28 |  | |  | | 9 | | |  | |  | | 19 | | |  | |  | |  |  |  |  |  |  |  |  |
|  | Separated | 28 |  | |  | | 6 | | |  | |  | | 22 | | |  | |  | |  |  |  |  |  |  |  |  |
|  | Single/never married | 434 |  | |  | | 190 | | |  | |  | | 243 | | |  | |  | |  |  |  |  |  |  |  |  |
|  | Widowed | 61 |  | |  | | 28 | | |  | |  | | 33 | | |  | |  | |  |  |  |  |  |  |  |  |
|  | Missing | 9 |  | |  | | 2 | | |  | |  | | 6 | | |  | |  | |  |  |  |  |  |  |  |  |
|  |  |  |  | |  | |  | | |  | |  | |  | | |  | |  | |  |  |  |  |  |  |  |  |
| **Type of residence** | |  |  | |  | |  | | |  | |  | |  | | |  | |  | |  |  |  |  |  |  |  |  |
|  | Large city | 212 |  | |  | | 97 | | |  | |  | | 115 | | |  | |  | |  |  |  |  |  |  |  |  |
|  | Rural | 105 |  | |  | | 38 | | |  | |  | | 66 | | |  | |  | |  |  |  |  |  |  |  |  |
|  | Small city or town | 256 |  | |  | | 80 | | |  | |  | | 175 | | |  | |  | |  |  |  |  |  |  |  |  |
|  | Suburb near large city | 249 |  | |  | | 151 | | |  | |  | | 98 | | |  | |  | |  |  |  |  |  |  |  |  |
|  | Missing | 1 |  | |  | |  | | |  | |  | |  | | |  | |  | |  |  |  |  |  |  |  |  |
|  |  |  |  | |  | |  | | |  | |  | |  | | |  | |  | |  |  |  |  |  |  |  |  |
| **Self-isolated in response to COVID-19** | | | |  | | | |  | | |  | | | |  | | |  | | | |  | |  | |  | |  |
|  | No | 421 | 115 | | 87 | | 118 | | | 27 | | 16 | | 302 | | | 87 | | 71 | |  |  |  |  |  |  |  |  |
|  | Somewhat | 330 | 312 | | 217 | | 204 | | | 111 | | 83 | | 124 | | | 201 | | 134 | |  |  |  |  |  |  |  |  |
|  | Yes | 53 | 325 | | 450 | | 35 | | | 196 | | 227 | | 18 | | | 128 | | 221 | |  |  |  |  |  |  |  |  |
|  | Missing | 19 | 72 | | 70 | | 9 | | | 32 | | 40 | | 10 | | | 38 | | 28 | |  |  |  |  |  |  |  |  |
|  |  |  |  | |  | |  | | |  | |  | |  | | |  | |  | |  |  |  |  |  |  |  |  |
| **Diagnosed or self-diagnosed with COVID-19** | | | | | |  | | |  | | | |  | | |  | | | |  | | |  | |  | |  | |
|  | No |  | 733 | | 718 | |  | | | 328 | | 312 | |  | | | 403 | | 404 | |  |  |  |  |  |  |  |  |
|  | Yes |  | 19 | | 36 | |  | | | 6 | | 14 | |  | | | 13 | | 22 | |  |  |  |  |  |  |  |  |
|  | Missing |  | 72 | | 70 | |  | | | 32 | | 40 | |  | | | 38 | | 28 | |  |  |  |  |  |  |  |  |
|  |  |  |  | |  | |  | | |  | |  | |  | | |  | |  | |  |  |  |  |  |  |  |  |
| **Recommendations about isolation** | | | |  | | | |  | | |  | | | |  | | |  | | | |  | |  | |  | |  |
|  | Public health agency | 252 | 544 | | 605 | | 173 | | | 276 | | 284 | | 78 | | | 268 | | 320 | |  |  |  |  |  |  |  |  |
|  | Personal doctor / physician | 24 | 43 | | 59 | | 15 | | | 21 | | 24 | | 9 | | | 21 | | 34 | |  |  |  |  |  |  |  |  |
|  | Family members / friends | 70 | 151 | | 157 | | 35 | | | 73 | | 76 | | 38 | | | 77 | | 80 | |  |  |  |  |  |  |  |  |
|  | News outlet | 176 | 314 | | 313 | | 126 | | | 172 | | 170 | | 50 | | | 142 | | 142 | |  |  |  |  |  |  |  |  |

Notes. Three participants did not report countries.

Frequency is reported at Time 2 and Time 3 after exclusion criteria are applied

APPENDIX B

**Directions**

A number of statements which people have used to describe themselves are given on the following pages. Read each statement and then select the appropriate button to indicate how you feel right now, that is, at this moment. There are no right or wrong answers. Do not spend too much time on any one statement but give the answer which seems to describe your present feelings best.

Not at all - Somewhat - Moderately so - Very much so


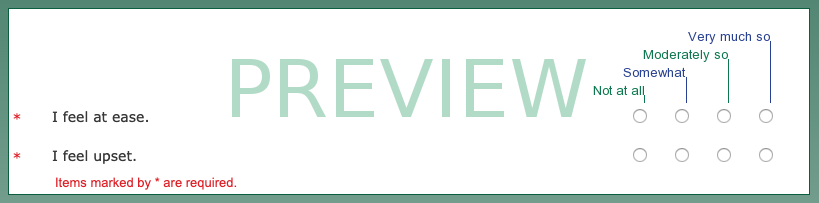


These sample items are not authorized for reuse or modification. We understand situations exist where you may want sample items such as academic, scientific or commentary purposes. If you are interested in the ability to reproduce or modify questions for these purposes, please contact info@mindgarden.com. Please understand that disclosing more than we have authorized will compromise the integrity and value of the instrument.

*SOM 1*

|  | Time 1 | Time 2 | Time 3 |
| --- | --- | --- | --- |
| **Of total sample** |  |  |  |
| % of those scoring more than mid-point ill-being | 20% (40%) | 20% (40%) | 20% (40%) |
| % of those spending 100% day alone to self-isolate* | 6% (23%) | 21% (40%) | 34% (47%) |
| **Broken down by % of time spent alone*** |  |  |  |
| Ill-being for those who spend less than 100% day alone | 2.93 (1.22) | 2.86 (1.26) | 2.78 (1.27) |
| Ill-being for those who spend 100% day alone | 3.12 (1.52) | 3.06 (1.39) | 2.97 (1.48) |
| ***This is a subset of total sample (Time 1: n = 379, Time 2: n = 636, Time 3: n = 666) that answered “yes” and “somewhat” to whether they self-isolate in response to COVID-19 | | | |

*SOM 2*

|  |  | Time 2 Ill-being | | | | | |  | Time 3 Ill-being | | | | | |
| --- | --- | --- | --- | --- | --- | --- | --- | --- | --- | --- | --- | --- | --- | --- |
|  |  | β | 95% CI | | partial *r*^2^ | *t* | *p* |  | β | 95% CI | | partial *r*^2^ | *t* | *p* |
|  |  |  | lower | upper |  |  |  |  |  | lower | upper |  |  |  |
| 1 | Gender^#^ | .06 | .01 | .12 | .01 | 2.23 | .026 |  | .05 | .00 | .10 | .00 | 1.79 | .074 |
| 2 | Age^#^ | -.15 | -.20 | -.09 | .03 | -5.08 | .000 |  | -.14 | -.19 | -.08 | .03 | -4.80 | .000 |
| 3 | Subjective health^#^ | -.14 | -.19 | -.08 | .03 | -4.79 | .000 |  | -.13 | -.19 | -.08 | .03 | -4.66 | .000 |
| 4 | In-person interactions^^^ | -.02 | -.08 | .03 | .00 | -.83 | .406 |  | -.04 | -.10 | .01 | .00 | -1.56 | .120 |
| 5 | Virtual interactions^^^ | -.07 | -.13 | -.02 | .01 | -2.50 | .013 |  | -.08 | -.13 | -.02 | .01 | -2.69 | .007 |
| 6 | Stressor checklist^^^ | .16 | .10 | .21 | .04 | 5.59 | .000 |  | .15 | .10 | .21 | .04 | 5.38 | .000 |
| 7 | Health Anxiety^^^ | .48 | .42 | .54 | .26 | 15.95 | .000 |  | .50 | .44 | .56 | .27 | 16.50 | .000 |
| 8 | COVID-19 diagnosis^^^ | .04 | -.02 | .09 | .00 | 1.38 | .169 |  | .03 | -.03 | .08 | .00 | 1.01 | .313 |
| 9 | Preference for solitude^#^ | .00 | -.06 | .06 | .00 | -.10 | .921 |  | -.02 | -.07 | .04 | .00 | -.50 | .618 |
| 10 | Identified motivation^#^ | -.14 | -.20 | -.08 | .03 | -4.65 | .000 |  | -.13 | -.19 | -.07 | .03 | -4.40 | .000 |
| 11 | External motivation | .17 | .11 | .22 | .05 | 5.92 | .000 |  | .14 | .09 | .20 | .03 | 5.03 | .000 |
